# Sex-Related Differences in Early In-Hospital Outcome (Technical Success and Complications) of Carotid Artery Stenting and Risk Factors of Carotid Artery Stenosis

**DOI:** 10.3390/diseases13090282

**Published:** 2025-09-01

**Authors:** Kinga Natalia Dudzińska, Paweł Muszyński, Joanna Kruszyńska, Konrad Bagiński, Maciej Kowalczuk, Konrad Nowak, Anna Tomaszuk-Kazberuk, Paweł Kralisz, Sławomir Dobrzycki, Marcin Kożuch

**Affiliations:** 1 Department of Cardiology, Lipidology and Internal Diseases, Medical University of Bialystok, Żurawia 14, 15-569 Bialystok, Poland; 2 Department of General and Experimental Pathology, Medical University of Bialystok, Mickiewicza 2C, 15-230 Bialystok, Poland; 3 Department of Invasive Cardiology, Medical University of Bialystok, M. Skłodowskiej-Curie 24A, 15-276 Bialystok, Poland

**Keywords:** carotid stenosis, atherosclerosis, sex-related differences, carotid artery stenting

## Abstract

Background/Objectives: Stroke and arteriosclerotic diseases remain the main challenge for global healthcare. Carotid artery procedures aim to restore blood flow through the carotid arteries to prevent embolic events. The most common techniques include carotid endarterectomy (CEA) and carotid artery stenting (CAS). The choice of intervention depends on the severity of stenosis, the patient’s overall condition and the presence of comorbidities. The personalized approach, which includes sex-related differences, is crucial in optimizing the outcome. Methods: Sex-related differences in atherosclerosis risk factors and early carotid artery stenting treatment outcomes were evaluated in 271 patients. The goal of the study was to asses sex-related differences in early outcome of CAS, including success rate and complications. Results: The only significant difference in classical arteriosclerosis risk factors included a higher occurrence of smoking among males. The technical success rate of carotid artery stenting was high (94.46%). The sex-related differences in CAS involve using smaller sizes of implanted stents in females. There was a high incidence of complications (mostly minor), predominantly among females. They had a significantly higher frequency of bleeding and hypotension. Blood pressure and BMI significantly influenced the odds of complications. Conclusions: Females undergoing CAS have a higher complication risk with a similar success rate.

## 1. Introduction

Atherosclerosis, classified as a chronic inflammatory and thrombotic condition, is considered a civilization disease due to its multifactorial etiology, involving both genetic and environmental factors [1,2]. One of the more frequent consequences of the pathological accumulation of atherosclerotic plaques is atherosclerotic stenosis of the internal carotid artery (ICA), which can lead to cerebral ischemia. The complications associated with this condition are particularly hazardous, not only due to the reduced blood flow to the central nervous system (as the ICA supplies over 65% of cerebral blood) but also because of the increased risk of plaque rupture, contributing to a higher likelihood of cerebral embolism [3]. It is estimated that 10% to 15% of all strokes result from thromboembolic events caused by ICA stenosis ranging from 50% to 99% [4].

Stroke, as defined by the World Health Organization (WHO), is the second leading cause of death globally and the foremost cause of disability. In Europe, 650,000 deaths annually are attributed to stroke [5].

Imaging of the carotid arteries is recommended in symptomatic patients and as a screening in high-risk patients (patients with severe coronary artery disease, or peripheral artery disease) [6]. Ultrasonography remains the primary imaging method. The North American Symptomatic Carotid Endarterectomy trial (NASCET) calculation using the lumen diameter in the lesion and in the distal segment of the artery, and the Doppler peak systolic and end-diastolic velocity criteria by the Society of Radiologists in Ultrasound (SRUCC), are currently recommended by guidelines and were also used in this study. In symptomatic patients, or when the regularization is planned, the other methods including computed tomography angiography (CTA) or magnetic resonance angiography (MRA) may be useful [6].

The gold standard for treating significant ICA stenosis remains surgical endarterectomy, which involves the removal of atherosclerotic plaques [7]. Alternatively, carotid artery stenting (CAS) may be considered, particularly in anatomical challenges, post-radiation stenosis, restenosis following previous surgeries or contraindications to surgery [8]. Studies showed that CAS has higher risk of periprocedural death or stroke; however, the risk of myocardial infarction is lower [9]. Additionally, in asymptomatic patients, optimal medical therapy (OMT) has similar outcomes to CAS in 5-year follow-ups [10]. The proper treatment choice should be made after careful evaluation of potential risks and benefits [11]. Currently available conservative treatment, including intensive hypolipemic treatment (with statins, ezetimibe or PCSK-9 inhibitors), as well as vascular dosing of anticoagulants, can be a successful alternative to invasive treatment [6]. Current guidelines recommend OMT for all patients with carotid artery stenosis. The invasive treatment is contraindicated for patients with total occlusion, or <50% stenosis in symptomatic patients and <60% stenosis in asymptomatic patients. The qualification for revascularization requires individual risk assessment and an individual patient approach involving a vascular team. In asymptomatic patients, it may be considered in 60–99% carotid stenosis (IIB recommendation); however, it should not be routinely performed in high-risk patients. In symptomatic patients in the presence of 50–69% carotid stenosis, revascularization should be considered (IIA recommendation), and in the presence of 70–99% carotid stenosis, it is recommended (I recommendation) [6].

Estrogen is responsible for cardioprotection in women, causing a few-year shift in atherosclerosis development. It is suggested that a low level of estrogen in the perimenopausal period may lead to the impairment of nitric oxide synthase functioning and to vasoconstriction [12]. Studies indicate that women have a higher risk of stroke during and after menopause, and men are more likely to develop larger atherosclerotic plaques. Moreover, they more frequently present with calcifications, lipid-rich necrotic cores, intraplaque hemorrhage and ulcerated plaque cores. One of the studies found that the male sex was independently associated with higher intraplaque hemorrhage and calcified plaque volumes ipsilateral to the stroke side, after adjusting for contralateral plaque volumes and BMI, indicating the association is not only due to larger vessel size in men [13,14,15]. The inflammatory markers also support greater plaque instability among males [16]. Additionally, the females’ cardiovascular risk can be influenced by sex-specific conditions, such as history of preeclampsia, pregnancy induced hypertension or gestational Diabetes Mellitus [17].

Studying sex differences in carotid artery atherosclerosis is vital, as the disease may progress differently in men and women, necessitating tailored diagnostic strategies and personalized treatment approaches. Moreover, differences in arterial compliance and the influence of hormonal factors can affect disease progression and treatment efficacy, highlighting the need for an individualized approach to patient care.

The primary goal of the study was to investigate sex-related differences in early outcomes of CAS, including success rate and complications. The secondary goal included evaluation of potential factors influencing outcomes and sex-related differences regarding atherosclerosis risk factors.

## 2. Materials and Methods

### 2.1. Data Collection

The analysis was performed retrospectively according to the data collected by the Invasive Cardiology Department of the Medical University of Bialystok, Poland, from 2007 to 2016. In total, 271 patients out of 11,155 total patients were included in the analysis. The study consisted of patients who had previously undergone coronary artery angiography who, due to high-risk features/symptoms, had carotid ultrasound, or who had been previously diagnosed with carotid artery stenosis and were referred for CAS.

The inclusion criteria required diagnosing occlusion and stenosis of precerebral arteries (I65 ICD-10 code) and performing carotid artery stenting (39.90 ICD 9 code).

**Figure 1:**
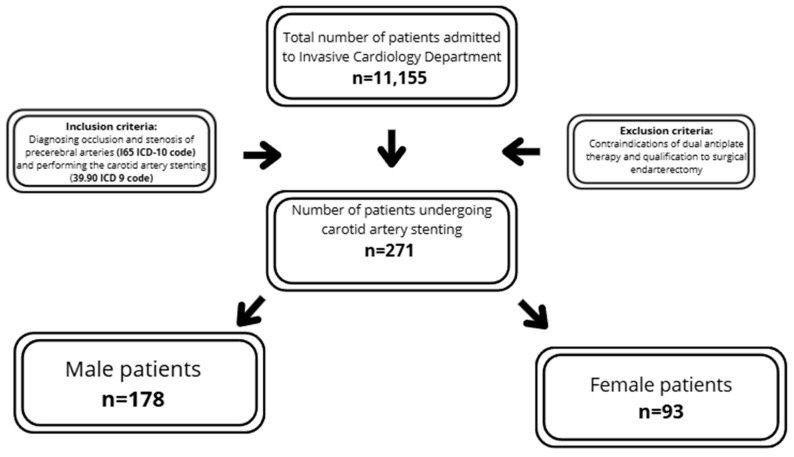
Flow-chart summarizing study population and inclusion/exclusion criteria.

The exclusion criteria encompassed contraindications to dual antiplatelet therapy and qualification for surgical endarterectomy. Each patient was assessed by a vascular team, including a vascular surgeon, angiologist and neurologist, according to European guidelines, and was qualified for percutaneous treatment (Figure 1). The summarized criteria are presented in Figure 1. The analysis included medical history, basic laboratory tests and clinical data. Carotid ultrasound was performed using iE33 xMatrix (Philips, Einthoven, The Netherlands) or ACUSON X300 (Siemens, Košice, Slovakia). Coronary/carotid angiographies were performed by Infinix CC- (Toshiba, Tokyo, Japan) or Infinix 8000C (Toshiba, Tokyo, Japan). The stenosis severity was assessed using NASCET criteria and peak systolic velocity-based evaluation [6]. When requested by the vascular team, additional imaging, including angio-CT or MRI, was performed. The early periprocedural results, including the success rate and early complications, were analyzed. The symptomatic assessment was not part of inclusion criteria; analysis was performed post-hoc after inclusion of the patients into the study. The evaluation of complications was limited to early in-hospital observation, and the diagnosis of post-CAS complication was performed by an attending physician, based on current guidelines.

### 2.2. Statistical Analysis

Continuous variables are expressed as median ± interquartile range (IQR). Categorical variables are shown as percentages (number of patients). The adequacy of all parameters to normal distribution was tested using the Kolmogorov–Smirnov test. Statistical tests used for analysis included Student’s *t*-test for parametric continuous variables; the Mann–Whitney U-test for non-parametric continuous categorical variables; and the Chi-squared test and odds ratio Altman calculation for categorical variables. The odds ratio comparison between sexes was formed as female vs. male. The statistics were performed using Statistica 13. The logistic regression was executed using Stata 18.0. Logistic regression coefficients present an outcome for a one-unit increase in the predictor variable. *p*-value ≤ 0.05 was considered as significant. The pairwise deletion approach was used in missing data curation. The Bonferroni correction was not included due to risk of type II errors and reduction of statistical power related to the large number of analyses performed on a few non-independent variables.

### 2.3. Ethics

The study was conducted under the Declaration of Helsinki and approved by the Institutional Review Board of the Medical University of Bialystok (R-I-002/167/2016). Informed consent was obtained from all subjects involved in this study.

### 2.4. Diagnostic Criteria for Disease Entities

The diseases were diagnosed based on medical history, previous medical records and/or the diagnostic criteria listed below.

Chronic kidney disease (CKD) was defined as kidney damage or decreased kidney function, lasting for ≥3 months, with one or both of the following: albuminuria (albumin-to-creatinine ratio ≥30 mg/g) or eGFR < 60 mL/min/1.73 m^2^.

Dyslipidemia was defined as serum concentration of triglycerides ≥1.7 mmol/L (≥150 mg/dL); HDL < 0.9 mmol/L (<35 mg/dL) in men and <1.0 mmol/L (<39 mg/dL) in women; or LDL above therapeutic goal.

Diabetes Mellitus was defined as 2 separate measurements of fasting blood glucose level of >126 mg/dL (7.0 mmol/L); a 2 h glucose level of >200 mg/dL (11.1 mmol/L) or higher during an oral glucose tolerance test (OGTT); or an HbA1c level of 6.5% (48 mmol/mol) or higher. Symptoms and random plasma glucose 200 mg/dL.

Prediabetes: fasting blood glucose 100–125 mg/dL (5.6 to 6.9 mmol/L), OGTT 140–199 mg/dL (7.8 to 11.0 mmol/L), HbA1C test: 5.7% to 6.4%.

Hyperfibrinogenemia was defined as plasma fibrinogen concentration >350 mg/L.

Hypertension was diagnosed when SBP ≥ 140 mmHg or DBP ≥ 90 mmHg.

Overweight was diagnosed with BMI ≥ 25 kg/m^2^.

Obesity was diagnosed with BMI ≥ 30 kg/m^2^.

Hyperuricemia was diagnosed when serum uric acid levels exceed 7.0 mg/dL in men and 6.0 mg/dL in women.

Metabolic syndrome—obesity: waist ≥ 88 cm (women), ≥102 cm (men) or BMI ≥ 30 kg/m^2^, plus at least 2 of the following: fasting glucose ≥ 100 mg/dL, 2 h glucose ≥ 140 mg/dL, HbA1c ≥ 5.7%, glucose-lowering treatment, non-HDL cholesterol ≥ 130 mg/dL, lipid-lowering treatment, office BP ≥ 130/85 mmHg, ambulatory BP ≥ 130/80 mmHg or antihypertensive treatment.

## 3. Results

### 3.1. Baseline Characteristics of Study Population

The study population comprised 271 patients with carotid artery stenosis, stratified into two subgroups based on sex: males (*n* = 178; 65.68%) and females (*n* = 93; 34.32%). The median age of the participants was 71 ±13.0 years. Additionally, the analyzed population demonstrated a high prevalence of atherosclerotic risk factors, including overweight (52.4%), hypertension (88.56%), Diabetes Mellitus (34.69%), dyslipidemia (66.79%) and chronic kidney disease (25.5%). A total of 60.15% of patients were asymptomatic, 25.46% suffered from stroke and 15.87% from TIA prior to the CAS procedure. Contralateral carotid occlusion occurred in 18.08% of subjects and bilateral carotid artery stenosis in 40.59% (Table 1).

There was no difference in age between males and females. Women had a lower body weight compared to men (72.00 ± 11.0 kg vs. 80.00 ± 17.0 kg). BMI in both studied groups was similar. However, there was a trend towards a higher percentage of women classified as overweight.

The prevalence of prediabetes and type 2 diabetes was identical in both sexes. No significant differences were observed in fasting blood glucose levels, but there was a trend towards higher glycated hemoglobin (HbA1c) levels in females (6.25 ± 0.9% vs. 6.05 ± 0.95%; *p* = 0.0609).

A statistically significant difference was observed in HDL-C [mg/dL] levels, with men having lower levels compared to women (47.00 ± 22.0 vs. 42.00 ± 12.0).

Hypertension was observed equally frequently in men and women. Pre-procedural systolic and diastolic blood pressure was identical between sexes. Post-procedural systolic blood pressure was also similar in both sexes, but males demonstrated higher values of diastolic blood pressure (64.00 ± 17.0 vs. 70.0 ± 17.0; *p* = 0.0013).

Prevalence of metabolic syndrome was the same in both sexes. Additionally, men were more likely to be active smokers (14.0% vs. 8.6%) and to report a history of smoking (13.98% vs. 22.9%) (Table 1).

|Significant coronary artery stenosis was more often present in males (71.0% vs. 83.7%; *p* = 0.0139). There was a trend toward higher presence of hyperuricemia among males, but statistical significance was not reached (15.05% vs. 23.03%; *p* = 0.0813).

In laboratory tests, males demonstrated higher levels of creatinine (0.83 ± 0.33 vs. 1.00 ± 0.35; *p* < 0.0001), eGFR (68.67 ± 27.0 vs. 75.27 ± 33.7; *p* < 0.0001), hemoglobin (12.60 ± 1.4 vs. 13.50 ± 1.70; *p* < 0.0001) and uric acid (5.60 ± 2.05 vs. 6.265 ± 2.1; *p* = 0.0045) (Table 1).

Beta-blockers were more frequently used by males than females (84.83% vs. 75.27%; *p* = 0.0540), as were angiotensin-converting enzyme inhibitors (ACE-I) (76.97% vs. 59.14%; *p* = 0.0022). However, angiotensin receptor blockers (ARB) were more frequently prescribed to females (24.73% vs. 12.92%; *p* = 0.0139) (Table 1).

### 3.2. Procedure Outcome

The success rate was a 94.46%. The complications occurred in 22.14% of patients.

**Figure 2:**
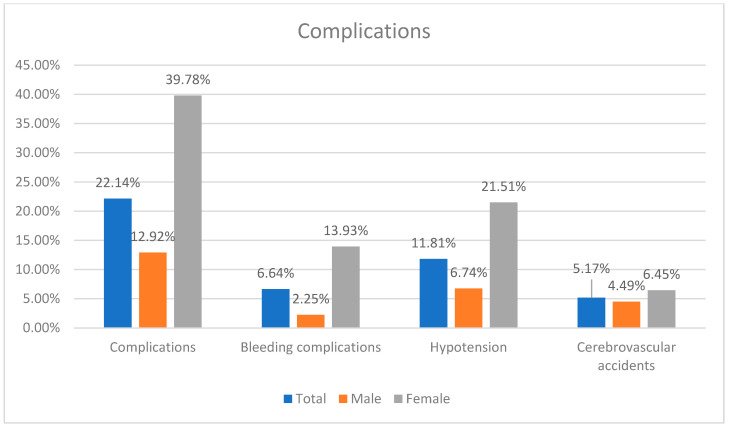
Summary of complication incidence.

There was no difference in success rate, cerebrovascular accidents, myocardial infarction and contrast-induced nephropathy between sexes. Females had higher risk of combined complications (OR: 4.4526; *p* < 0.0001), bleeding complications (OR: 5.6225; *p* = 0.0015) and hypotension (OR: 3.7900; *p* = 0.0007) (Table 2) (Figure 2).

Statistically significant differences in stent dimensions were observed between sexes, with male patients receiving stents of greater minimal width (7.0 ± 0.89 mm vs. 6.0 ± 0.62 mm), maximal width (9.0 ± 1.03 mm vs. 8.0 ± 0.58 mm) and length (40.0 ± 7.95 mm vs. 30.0 ± 5.07 mm) than female patients (Table 3).

### 3.3. The Analysis of Pre-Procedure Risk Factors of Complications Using Multivariable Logistic Regression

The multivariable logistic regression showed that a higher SBP before the procedure decreased the risk of combined complications (OR: 0.97; *p* = 0.005). A similar trend was found with hemoglobin concentration, but statistical significance was not reached (OR: 0.78; *p* = 0.063). The sex-specific results suggest a lower risk of compilations in females with higher SBP (OR: 0.97; *p* = 0.029) and lower platelet count (PLT) (OR: 1.01; *p* = 0.04), and in males with lower fibrinogen (OR: 1.01; *p* = 0.045). There was a tendency for comparable results in males regarding SBP, but statistical significance was not reached (OR: 0.97; *p* = 0.059). Hypertension decreased risk of complications (OR: 0.40), an effect that was especially noticeable in men (OR: 0.19). In females, one of the most significantly influential factors was being asymptomatic (OR: 4.14) (Table 4).

The odds of cerebrovascular complications increased with WBCs (OR: 1.51; *p* = 0.017) and tend to increase with age (OR: 1.11; *p* = 0.067). In males, the increase in fibrinogen causes a slight increase in the risk of cerebrovascular complications (OR: 1.01; *p* = 0.041) (Table 5).

**Figure 3:**
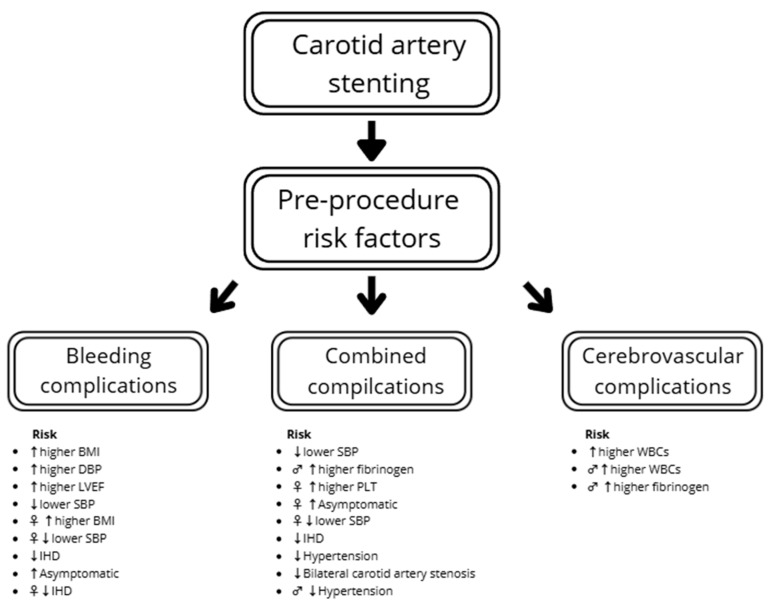
Combined risk factors of complications. Legend: ♂ in male, ♀ in female, ↑ increased risk, ↓ decreased risk. BMI—body mass index; SBP—systolic blood pressure; DBP—diastolic blood pressure; IHD—ischemic heart disease; LVEF—left ventricle ejection fraction; PLT—platelet count; WBCs—white blood cells.

In the total study population, increased BMI (OR: 1.29; *p* = 0.006), DBP before the procedure (OR: 1.06; *p* = 0.038) and LVEF (OR: 1.10; *p* = 0.025) were connected with an increased risk of bleeding complications. The patients with ischemic heart disease had lower bleeding risk. However, none of the factors were significant in males, with only a tendency in PLT and anticoagulation. In females, ischemic heart disease, BMI and SBP significantly influence the risk of bleeding complications (Table 6). The summarized results regarding complications are presented in Figure 3.

## 4. Discussion

### 4.1. Sex-Related Differences in Complications After Carotid Artery Stenting

Pre-procedural risk factors play a key role in predicting the type and frequency of complications after carotid artery stenting, including bleeding, cerebrovascular and combined complications. Some studies suggest that outcomes after CAS are similar for males and females [18,19]. However, in symptomatic patients in the CREST trial, the females had a higher combined primary endpoint in the periprocedural period (9.2% vs. 5.4%, *p* = 0.04) [20]. In our study, females had a higher risk of combined complications, bleeding complications and hypotension.

Saleh et al. showed that hemodynamic instability can occur even in 31.2% of patients who underwent CAS. The risk factors predicting this event included hypertension, symptomatic carotid lesions, right-sided lesions, hyperechoic/calcified plaques and longer lesions [21]. In another study, 45.3% of patients experienced hemodynamic instability, and risk factors were smoking and a large-diameter balloon (>4 mm) used during the procedure [22]. In our study, hypotension after the procedure was present in 11.81%, and the higher SBP before the procedure could have a protective effect regarding the CAS complications. In a study by Faateh et al., fragile patients had higher risk of complications after CAS, especially when asymptomatic [23]. Our study did not show any correlation between age and complications, even though 60.15% of patients were asymptomatic. Additionally, females had higher risk of hypotension (OR: 3.7900) and requiring catecholamine infusion (OR: 3.1481).

In other studies, vascular complications occurred in around 5% of patients [24]. The bleeding complications in our study occurred in 6.64%, predominantly in females (OR: 5.6225). The bleeding complications significantly increase risk of readmission after CAS; thus, investigation of risk factors and prevention tools is crucial [24,25]. In our study, the leading risk factors for bleeding were higher BMI, DBP and LVEDF, and lower SBP. Additionally, asymptomatic females were at higher risk of bleeding complications. BMI was a risk factor for bleeding complications only for females, which could be influenced by different obesity phenotypes between sexes and dominant femoral approach. The lower incidence of bleeding complications among females with ischemic heart disease remains unclear and should be subject to further analysis.

Additionally, there may be a trend toward a higher rate of neurological complications among symptomatic females undergoing CAS and a higher rate of MI among asymptomatic patients [14]. Kramer et al. showed that in asymptomatic patients there are no significant differences in occurrence of stroke [14]. The opposite outcome was shown by Etkin et al.: symptomatic patients had a higher rate of periprocedural MI and there were no differences in asymptomatic patients [26]. Erben et al. showed risk of TIA/stroke was higher in females, but in long-term follow-up mortality was higher in males [27]. However, in our study there was no sex-related difference in occurrence of MI and cerebrovascular events.

The higher risk of complication in asymptomatic females should influence future guidelines to include sex-related differences in the decision-making process.

Our study, in comparison to those previously published, not only assessed the outcome of the CAS procedure, but also searched for potential risk factors of poor outcomes with specific consideration to sex-related differences. Previously, published studies found a higher bleeding and hypotension risk among females. However, our study allowed for the identification of potential risk factors influencing this finding, and should be verified in prospective studies [28,29].

### 4.2. Sex-Related Differences in Risk Factors and Prevention of Atherosclerosis

Males have higher risk of atherosclerosis. Coronary atherosclerosis (OR: 2.75 95% CL: 2.53–2.99) and carotid plaque (OR: 1.57 95% CL: 1.45–1.70) occur more frequently in males [30]. However, the atherosclerosis-related diseases such as coronary artery disease or stroke are leading causes of deaths in both sexes, and are a significant global health problem. Both males and females should be equally included in prevention by intensive risk factor control. The European Society of Cardiology guidelines regarding CVD prevention state that sex-specific awareness campaigns have already been started, but importance of heart disease in women should remain a crucial subject of future studies [17]. Research suggests female patients with arteriosclerosis use aspirin therapy less frequently compared to the male population, and are prescribed lower doses of statins or less-intensive statins compared to men [12]. The stroke incidence is lower in females (3.9 vs. 4.4 per 1000 person-years), but still remains a large public health problem and one of the main goals for prevention. Hypertension and smoking is affecting the risk of stroke in both sexes; however, HDL-C has a protective effect only among females [31]. In our study, active smoking or history of smoking was more prevalent among males, and HDL-C cholesterol was higher among females. Furthermore, there are sex-related differences in carotid atherosclerosis. Males have higher risk of larger plaque, with calcifications, lipid-rich necrotic core, intraplaque hemorrhage or ulcer [13]. Females have less-typical symptoms related to stroke or carotid arteriosclerosis, which can contribute to misdiagnosis [32]. In our study, there was no significant difference in target vessel stenosis or symptomology. Additionally, women had lower prevalence of significant coronary artery lesions, as well as a tendency towards lower rate of PAD.

## 5. Study Limitations

One of the primary limitations of this study is its retrospective, observational design. Retrospective studies inherently suffer from biases related to data collection, as they rely on pre-existing medical records. This can lead to inconsistencies in data quality, missing information and an inability to control for confounding variables effectively.

Additionally, the study population was exclusively composed of individuals from Eastern Europe, and all participants were Caucasian. This demographic homogeneity limits the generalizability of the findings to other ethnic groups, as genetic, socioeconomic and environmental factors may influence disease presentation, treatment response and clinical outcomes. Ethnic variability in cardiovascular risk factors and treatment efficacy is well-documented; thus, a more diverse study population would have enhanced the external validity of the findings.

Without longitudinal monitoring, key clinical endpoints, such as survival rates and long-term major adverse cardiac and cerebrovascular events (MACCEs), remain unexamined. The absence of follow-up data prevents an assessment of the durability and effectiveness of the studied interventions over time, which is crucial for understanding the real-world impact of medical treatments and disease progression.

Future research should aim for prospective, multicenter studies with more diverse populations and comprehensive data collection to enhance the applicability and reliability of the findings.

## 6. Conclusions

Despite similar exposure to classical risk factors, females less frequently had significant coronary artery disease. The sex-related differences in CAS include the smaller size of stents and a higher rate of complications in females, with similar success rates. Blood pressure and BMI remain the most critical factors influencing complications. Asymptomatic females had higher risk of complications.

## Figures and Tables

**Table 1:** Baseline characteristics.

Baseline Characteristics	Total *n* = 271	Male *n* = 178	Female *n* = 93	*p*-Value
Sex (male)	65.68% (178)	-	-	-
Age (above 75 y.)	34.3% (93)	34.8% (62)	33.3% (31)	0.8049
Age [y.]	71.00 ± 13.0	72.00 ± 13.0	71.00 ± 13.0	0.5842
Symptomology				
Asymptomatic	60.15% (163)	57.30% (102)	65.59% (61)	0.4459
Stroke	25.46% (69)	27.53% (49)	21.51% (20)	
TIA	15.87% (43)	16.29% (29)	15.05% (14)	
Target vessel				
RICA	57.93% (157)	59.55% (106)	54.84% (51)	0.4559
LICA	42.07% (114)	40.45% (72)	45.16% (42)	
Target vessel stenosis				
RICA	0.80 ± 0.34	0.80 ± 0.35	0.80 ± 0.32	0.2234
LICA	0.80 ± 0.36	0.80 ± 0.35	0.70 ± 0.36	0.076
Contralateral carotid occlusion	18.08% (49)	20.78% (37)	12.90% (12)	0.109
Bilateral carotid artery stenosis	40.59% (110)	44.38% (79)	33.33% (31)	0.079
Risk factor				
Overweight	52.4% (142)	54.5% (97)	48.4% (45)	0.098
Obesity	29.5% (80)	28.7% (51)	31.2% (29)	
Weight [kg]	77.00 ± 17.0	80.00 ± 17.0	72.00 ± 11.0	<0.0001
Height [cm]	168.00 ± 13.0	170.50 ± 8.0	160.00 ± 8.0	<0.0001
BMI	28.00 ± 4.00	28.00 ± 4.0	28.00 ± 5.0	0.8695
BSA	1.87 ± 0.26	1.94 ± 0.23	1.74 ± 0.14	<0.0001
Diabetes Mellitus	34.69% (94)	34.83% (62)	34.41% (32)	0.9436
Prediabetes	9.2% (25)	9.6% (17)	8.6% (8)	0.7973
Glucose [mg/dL]	105.50 ± 37.0	104.00 ± 38.0	109.50 ± 36.0	0.5178
HBA1C [%]	6.20 ± 1.1	6.05 ± 0.95	6.25 ± 0.9	0.0609
Dyslipidemia	66.79% (181)	63.48% (113)	73.12% (68)	0.1098
TC [mg/dL]	152.00 ± 46.00	150.00 ± 41.0	157.00 ± 46.0	0.0905
LDL-C [mg/dL]	86.00 ± 38.00	86.00 ± 38.0	87.00 ± 41.0	0.8132
HDL-C [mg/dL]	44.00 ± 14.00	42.00 ± 12.0	47.00 ± 22.0	<0.0001
TGs [mg/dL]	111.00 ± 66.00	113.00 ± 74.0	111.00 ± 54.0	0.7627
Hypertension	88.56% (240)	88.76% (158)	88.17% (82)	0.8848
SBP before [mmHg}	134.50 ± 30.0	134.50 ± 29.0	134.00 ± 39.5	0.4433
DBP before [mmHg]	71.50 ± 14.0	73.50 ± 14.0	70.00 ± 16.0	0.4318
SBP after [mmHg}	122.00 ± 27.0	123.50 ± 25.0	120.00 ± 30.0	0.3491
DBP after [mmHg]	68.0 ± 17.0	70.0 ± 17.0	64.00 ± 17.0	0.0013
Metabolic syndrome	30.9% (67)	21.9% (39)	30.1% (28)	0.1374
Active smoking	12.2% (33)	14.0% (25)	8.6% (8)	0.0001
Smoking history	27.7% (75)	22.9% (62)	13.98% (13)	
COPD	5.5% (15)	6.7% (12)	3% (3)	0.2295
Chronic coronary syndrome	95.2% (258)	97.2% (173)	91.4% (85)	0.1381
Significant coronary artery stenosis	79.3% (215)	83.7% (149)	71.0% (66)	0.0139
Past MI	36.9% (100)	39.9% (69)	33.3% (31)	0.3790
PAD	40.96% (111)	44.94% (80)	33.33% (31)	0.0650
Chronic kidney disease	25.5% (69)	24.2% (43)	28.0% (26)	0.3839
Creatinine [mg/dL}	0.96 ± 0.35	1.00 ± 0.35	0.83 ± 0.33	<0.0001
eGFR [mL/min/1.73 m^2^]	73.23 ± 30.3	75.27 ± 33.7	68.67 ± 27.0	<0.0001
Anemia	25.83% (70)	25.28% (45)	26.88% (25)	0.7746
Hemoglobin [g/dL]	13.10 ± 1.8	13.50 ± 1.70	12.60 ± 1.4	<0.0001
Hyperuricemia	20.30% (55)	23.03% (41)	15.05% (14)	0.0813
Uric acid [mg/dL]	5.97 ± 2.04	6.265 ± 2.1	5.60 ± 2.05	0.0045
Hyperfibrinogenemia	44.28% (120)	41.01% (73)	50.54% (47)	0.1339
Fibrinogen [mg/dL]	385.5 ± 103.0	382.5 ± 104.0	401.5 ± 108.5	0.2003
TSH	0.78 ± 1.1	0.70 ± 1.1	0.90 ± 0.84	0.1621
Atrial fibrillation	15.9% (43)	15.7% (28)	16.1% (15)	0.9333
Medical treatment				
Beta-blockers	81.55% (221)	84.83% (151)	75.27% (70)	0.0540
ARB	16.97% (46)	12.92% (23)	24.73% (23)	0.0139
ACE-I	70.85% (192)	76.97% (137)	59.14% (55)	0.0022
Statins	90.04% (244)	91.01% (162)	88.17% (82)	0.4587
Anticoagulation	11.07% (30)	11.80% (21)	9.68% (9)	0.5973

BMI—body mass index; BSA—body surface area; TC—total cholesterol; LDL-C—low-density lipoprotein cholesterol; HDL-C—high-density lipoprotein cholesterol; TGs—triglycerides; SBP—systolic blood pressure; DBP—diastolic blood pressure; COPD—chronic obstructive pulmonary disease; PAD—peripheral artery disease; MI—myocardial infarction; TSH—thyroid-stimulating hormone; eGFR—estimated glomerular filtration rate; ARB—angiotensin receptor blockers; ACE-I—angiotensin-converting enzyme; TIA—transient ischemic attack; RICA—right internal carotid artery; LICA—left internal carotid artery.

**Table 2:** Procedure outcome.

Outcome	Total *n* = 271	Male *n* = 178	Female *n* = 93	OR	CL 95%	*p*-Value
Technical success rate	94.46% (256)	93.82% (167)	95.70% (89)	1.4656	0.4535 to 4.7360	0.5230
Complications	22.14% (60)	12.92% (23)	39.78% (37)	4.4526	2.4351 to 8.1419	<0.0001
Bleeding complications	6.64% (18)	2.81% (5)	13.98% (13)	5.6225	1.9384 to 16.3087	0.0015
Bleeding from access site	3.69% (10)	2.25% (4)	6.45% (6)	3.0000	0.8249 to 10.9102	0.0954
Hematoma in access site	4.06% (11)	1.12% (2)	9.68% (9)	9.4286	1.9930 to 44.6046	0.0047
Blood transfusion	1.48% (4)	0.56% (1)	3.23% (3)	5.9000	0.6051 to 57.5323	0.1266
Hypotension	11.81% (32)	6.74% (12)	21.51% (20)	3.7900	1.7604 to 8.1593	0.0007
Catecholamine infusion	7.38% (20)	4.49% (8)	12.90% (12)	3.1481	1.2385 to 8.0024	0.0160
Cerebrovascular accidents	5.17%% (14)	4.49% (8)	6.45% (6)	1.4655	0.4929 to 4.3571	0.4917
TIA	2.95% (8)	2.25% (4)	4.30% (4)	1.9551	0.4777 to 8.0020	0.3511
Stroke	2.21% (6)	2.25% (4)	2.15% (2)	0.9560	0.1718 to 5.3190	0.9591
Myocardial infarction	0.00% (0)	0.00% (0)	0.00% (0)	1.9091	0.0376 to 96.9897	0.7470
CIN	1.48% (4)	1.12% (2)	2.15% (2)	1.9341	0.2680 to 13.9558	0.5130

TIA—transient ischemic attack; CIN—contrast-induced nephropathy.

**Table 3:** Procedure details.

Procedure Details		Total *n* = 271	Male *n* = 178	Female *n* = 93	*p*-Value
Stent type	Xact	74.54% (202)	72.47% (129)	78.49% (73)	0.2426
	Acculink	12.92% (35)	14.04% (25)	10.75% (10)	
	Cristallo	0.74% (2)	0.00% (0)	2.15% (2)	
	Abbott Vascular	1.11% (3)	1.69% (3)	0.00% (0)	
	Multilink	0.00% (0)	0.00% (0)	0.00% (0)	
	Jaguar	0.37% (1)	0.56% (1)	0.00% (0)	
	Sinus-Carotid-Conical-RX	3.32% (9)	3.93% (7)	2.15% (2)	
	Roadsaver	0.37% (1)	0.00% (0)	1.08% (1)	
Stent properties [mm]	Stent minimal width	7.0 ± 0.81	7.0 ± 0.89	6.0 ± 0.62	0.0030
	Stent maximal width	9.0 ± 0.91	9.0 ± 1.03	8.0 ± 0.58	0.0015
	Stent length	40.0 ± 7.23	40.0 ± 7.95	30.0 ± 5.07	0.0008
Vascular access	Femoral access	92.62% (251)	93.26% (166)	91.40% (85)	0.5783
	Radial access	7.38% (20)	6.74% (12)	8.60% (8)	

**Table 4:** The multivariable logistic regression and Altman calculation analyzing the impact of various factors on the complications of carotid artery stenting with subanalysis including the sex-related differences.

Complications	Factors	Odds Ratio	SE	*p* Value	CL 95%
Total	Age	1.03	0.02	0.215	0.98–1.07
	BMI	1.05	0.05	0.302	0.95–1.16
	SBP before	0.97	0.01	0.005	0.95–0.99
	DBP before	1.03	0.02	0.158	0.99–1.06
	Hemoglobin	0.78	0.10	0.063	0.60–1.01
	PLT	1.00	0.003	0.985	0.99–1.01
	WBCs	1.00	0.10	0.982	0.82–1.23
	Fibrinogen	1.00	0.002	0.193	1.00–1.01
	eGFR	1.00	0.02	0.701	0.99–1.02
	LVEF	1.02	0.012	0.273	0.99–1.05
	IHD	0.41	-	0.007	0.22–0.78
	Past MI	0.99	-	0.966	0.54–1.79
	AF	1.48	-	0.295	0.71–3.10
	Anticoagulation	1.09	-	0.858	0.44–2.67
	Hypertension	0.40	-	0.021	0.18–0.87
	Dyslipidemia	0.90	-	0.739	0.49–1.65
	Diabetes Mellitus	1.02	-	0.954	0.56–1.86
	Asymptomatic	1.69	-	0.094	0.91–3.14
	Contralateral carotid occlusion	0.54	-	0.162	0.23–1.28
	Bilateral carotid artery stenosis	0.34	-	<0.001	0.18–0.63
Male	Age	1.07	0.046	0.130	0.98–1.16
	BMI	0.99	0.08	0.920	0.84–1.17
	SBP before	0.97	0.02	0.059	0.94–1.00
	DBP before	1.04	0.03	0.268	0.97–1.10
	Hemoglobin	0.95	0.15	0.751	0.70–1.30
	PLT	1.00	0.005	0.985	0.99–1.01
	WBCs	1.13	0.18	0.430	0.83–1.54
	Fibrinogen	1.01	0.003	0.045	1.0001–1.01
	eGFR	1.02	0.02	0.264	0.99–1.05
	LVEF	0.99	0.02	0.607	0.95–1.03
	IHD	0.66	-	0.451	0.22–1.95
	Past MI	1.53	-	0.342	0.64–3.70
	AF	1.59	-	0.400	0.54–4.72
	Anticoagulation	1.14	-	0.842	0.31–4.23
	Hypertension	0.16	-	<0.001	0.06–0.45
	Dyslipidemia	1.09	-	0.853	0.44–2.73
	Diabetes Mellitus	1.52	-	0.353	0.63–3.70
	Asymptomatic	0.747	-	0.515	0.31–1.80
	Contralateral carotid occlusion	0.534	-	0.333	0.15–1.90
	Bilateral carotid artery stenosis	0.63	-	0.324	0.25–1.57
Female	Age	1.05	0.037	0.186	0.98–1.12
	BMI	1.08	0.10	0.397	0.91–1.28
	SBP before	0.97	0.01	0.029	0.95–0.997
	DBP before	1.02	0.02	0.405	0.97–1.07
	Hemoglobin	0.72	0.23	0.30	0.38–1.34
	PLT	1.01	0.01	0.04	1.001–1.02
	WBCs	0.94	0.16	0.727	0.67–1.32
	Fibrinogen	0.999	0.004	0.857	0.99–1.01
	eGFR	1.01	0.02	0.429	0.98–1.05
	LVEF	1.02	0.03	0.525	0.96–1.08
	IHD	0.43	-	0.069	0.18–1.07
	Past MI	0.76	-	0.550	0.31–1.86
	AF	1.40	-	0.553	0.46–4.26
	Anticoagulation	1.24	-	0.764	0.31–4.94
	Hypertension	1.18	-	0.805	0.32–4.35
	Dyslipidemia	0.50	-	0.148	0.20–1.27
	Diabetes Mellitus	0.71	-	0.441	0.29–0.71
	Asymptomatic	4.14	-	0.004	1.57–10.96
	Contralateral carotid occlusion	0.67	-	0.528	0.19–2.35
	Bilateral carotid artery stenosis	0.64	-	0.337	0.26–1.59

BMI—body mass index; SBP—systolic blood pressure; DBP—diastolic blood pressure; IHD—ischemic heart disease; Past MI—past myocardial infarction; AF—atrial fibrillation; PLT—platelet count; WBCs—white blood cells; LVEF—left ventricle ejection fraction.

**Table 5:** The multivariable logistic regression and Altman calculation analyzing the impact of various factors on the cerebrovascular complications of carotid artery stenting with subanalysis including the sex-related differences.

Cerebrovascular Complications	Factors	Odds Ratio	SE	*p* Value	CL 95%
Total	Age	1.11	0.06	0.067	0.99–1.23
	BMI	1.07	0.12	0.536	0.86–1.33
	SBP before	1.00	0.02	0.846	0.96–1.03
	DBP before	1.01	0.04	0.856	0.94–1.08
	Hemoglobin	0.82	0.18	0.373	0.53–1.27
	PLT	0.99	0.01	0.177	0.98–1.00
	WBCs	1.51	0.26	0.017	1.08–2.11
	Fibrinogen	1.01	0.004	0.111	0.999–1.01
	eGFR	1.01	0.02	0.555	0.97–1.05
	LVEF	1.01	0.03	0.685	0.95–1.10
	IHD	0.70	-	0.557	0.21–2.29
	Past MI	0.41	-	0.175	0.11–1.49
	AF	1.93	-	0.240	0.64–5.78
	Anticoagulation	1.25	-	0.774	0.27–5.84
	Hypertension	0.81	-	0.813	0.18–3.87
	Dyslipidemia	0.99	-	0.992	0.33–3.00
	Diabetes Mellitus	2.15	-	0.127	0.81–5.75
	Asymptomatic	1.36	-	0.589	0.45–4.08
	Contralateral carotid occlusion	0.68	-	0.621	0.15–3.12
	Bilateral carotid artery stenosis	0.51	-	0.261	0.16–1.65
Male	Age	1.15	0.11	0.158	0.95–1.38
	BMI	1.09	0.20	0.642	0.76–1.55
	SBP before	1.02	0.03	0.502	0.96–1.08
	DBP before	0.99	0.06	0.828	0.87–1.11
	Hemoglobin	0.87	0.30	0.692	0.44–1.72
	PLT	0.97	0.01	0.053	0.95–1.00
	WBCs	2.01	0.62	0.023	1.10–3.66
	Fibrinogen	1.01	0.01	0.041	1.0004–1.02
	eGFR	1.03	0.03	0.313	0.97–1.092
	LVEF	1.01	0.04	0.752	0.93–1.10
	IHD	0.30	-	0.114	0.07–1.34
	Past MI	0.09	-	0.094	0.01–1.51
	AF	3.48	-	0.102	0.78–15.49
	Anticoagulation	2.65	-	0.253	0.50–14.07
	Hypertension	0.36	-	0.225	0.07–1.89
	Dyslipidemia	0.97	-	0.953	0.22–4.14
	Diabetes Mellitus	3.30	-	0.110	0.76–14.31
	Asymptomatic	0.70	-	0.622	0.17–2.89
	Contralateral carotid occlusion	1.29	-	0.764	0.25–6.65
	Bilateral carotid artery stenosis	0.74	-	0.690	0.17–3.21
Female	Age	1.17	0.14	0.196	0.92–1.49
	BMI	1.03	0.18	0.862	0.73–1.46
	SBP before	0.97	0.03	0.228	0.92–1.02
	DBP before	1.04	0.06	0.509	0.93–1.16
	Hemoglobin	0.37	0.23	0.114	0.11–1.26
	PLT	1.01	0.01	0.492	0.98–1.03
	WBCs	1.28	0.37	0.398	0.72–2.27
	Fibrinogen	1.01	0.01	0.387	0.99–1.02
	eGFR	0.98	0.04	0.531	0.90–1.06
	LVEF	1.01	0.07	0.930	0.89–1.14
	IHD	2.60	-	0.387	0.30–22.69
	Past MI	1.55	-	0.581	0.33–7.42
	AF	0.86	-	0.890	0.10–7.68
	Anticoagulation	0.06	-	0.055	0.003–1.06
	Hypertension	2.28	-	0.580	0.12–42.76
	Dyslipidemia			0.917	0.17–5.04
	Diabetes Mellitus	1.47	-	0.626	0.31–7.03
	Asymptomatic	3.81	-	0.225	0.44–33.07
	Contralateral carotid occlusion	0.39	-	0.530	0.02–7.30
	Bilateral carotid artery stenosis	0.31	-	0.283	0.04–2.66

BMI—body mass index; SBP—systolic blood pressure; DBP—diastolic blood pressure; IHD—ischemic heart disease; Past MI—past myocardial infarction; AF—atrial fibrillation; PLT—platelet count; WBCs—white blood cells; LVEF—left ventricle ejection fraction.

**Table 6:** The multivariable logistic regression and Altman calculation analyzing the impact of various factors on the bleeding complications of carotid artery stenting with subanalysis including the sex-related differences.

Bleeding Complications	Factors	Odds Ratio	SE	*p* Value	CL 95%
Total	Age	1.02	0.03	0.549	0.96–1.08
	BMI	1.29	0.12	0.006	1.08–1.56
	SBP before	0.97	0.02	0.027	0.937–0.996
	DBP before	1.06	0.03	0.038	1.00–1.12
	Hemoglobin	1.08	0.18	0.312	0.51–1.24
	PLT	1.01	0.01	0.130	1.00–1.02
	WBCs	0.93	0.17	0.690	0.65–1.33
	Fibrinogen	1.00	0.004	0.601	0.99–1.01
	eGFR	0.97	0.01	0.056	0.94–1.00
	LVEF	1.10	0.04	0.025	1.01–1.19
	IHD	0.29	-	0.014	0.11–0.78
	Past MI	0.64	-	0.41	0.22–1.85
	AF	2.22	-	0.15	0.75–6.58
	Anticoagulation	1.67-	-	0.438	0.46–6.16
	Hypertension	0.42	-	0.148	0.13–1.36
	Dyslipidemia	1.80	-	0.312	0.58–5.64
	Diabetes Mellitus	0.52	-	0.258	0.17–1.62
	Asymptomatic	3.57	-	0.049	1.01–12.65
	Contralateral carotid occlusion	0.90	-	0.866	0.25–3.22
	Bilateral carotid artery stenosis	0.71	-	0.510	0.26–1.96
Male	Age	1.08	0.10	0.415	0.90–1.29
	BMI	1.23	0.23	0.276	0.85–1.78
	SBP before	0.99	0.04	0.797	0.92–1.06
	DBP before	1.04	0.06	0.549	0.92–1.16
	Hemoglobin	1.02	0.16	0.881	0.75–1.40
	PLT	1.01	0.01	0.093	1.00–1.03
	WBCs	0.82	0.29	0.568	0.41–1.64
	Fibrinogen	1.00	0.01	0.831	0.99–1.02
	eGFR	0.98	0.03	0.567	0.93–1.04
	IHD	2.25	-	0.587	0.12–41.72
	Past MI	2.43	-	0.337	0.40–14.94
	AF	3.77	-	0.157	0.60–23.67
	Anticoagulation	5.40	-	0.074	0.85–34.42
	Hypertension	0.49	-	0.537	0.05–4.65
	Dyslipidemia	6.64	-	0.203	0.36–122.06
	Diabetes Mellitus	1.24	-	0.820	0.20–7.59
	Asymptomatic	8.24	-	0.156	0.45–151.29
	Contralateral carotid occlusion	0.95	-	0.965	0.10–8.78
	Bilateral carotid artery stenosis	0.83	-	0.842	0.14–5.10
	LVEF	1.11	0.10	0.256	0.93–1.32
Female	Age	1.04	0.04	0.323	0.96–1.12
	BMI	1.33	0.17	0.027	1.03–1.71
	SBP before	0.96	0.02	0.041	0.928–0.998
	DBP before	1.06	0.03	0.080	0.99–1.13
	Hemoglobin	0.98	0.15	0.908	0.72–1.34
	PLT	1.00	0.01	0.764	0.99–1.02
	WBCs	1.07	0.24	0.761	0.69–1.67
	Fibrinogen	0.995	0.01	0.404	0.98–1.01
	eGFR	0.98	0.02	0.519	0.94–1.03
	LVEF	1.06	0.06	0.301	0.95–1.19
	IHD	0.19	-	0.008	0.06–0.66
	Past MI	0.32	-	0.156	0.07–1.54
	AF	1.70	-	0.467	0.41–7.10
	Anticoagulation	0.67	-	0.712	0.08–5.75
	Hypertension	0.37	-	0.189	0.08–1.63
	Dyslipidemia	0.80	-	0.734	0.22–2.88
	Diabetes Mellitus	0.30	-	0.137	0.06–1.46
	Asymptomatic	2.15	-	0.272	0.55–8.45
	Contralateral carotid occlusion	1.25	-	0.787	0.24–6.51
	Bilateral carotid artery stenosis	0.86	-	0.810	0.24–3.04

BMI—body mass index; SBP—systolic blood pressure; DBP—diastolic blood pressure; IHD—ischemic heart disease; Past MI—past myocardial infarction; AF—atrial fibrillation; PLT—platelet count; WBCs—white blood cells; LVEF—left ventricle ejection fraction.

## Data Availability

The data presented in this study are available on request from the corresponding author.
